# How Engaged Are Family Physicians in Addressing the Social Determinants of Health? A Survey Supporting the American Academy of Family Physician's Health Equity Environmental Scan

**DOI:** 10.1089/heq.2019.0022

**Published:** 2019-08-23

**Authors:** Kevin A. Kovach, Kathy Reid, Jené Grandmont, Danielle Jones, Julie Wood, Bellinda Schoof

**Affiliations:** ^1^ American Academy of Family Physicians, Leawood, Kansas.; ^2^ HealthLandscape, American Academy of Family Physicians, Cincinnati, Ohio.

**Keywords:** social determinants of health, family physician, health equity

## Abstract

**Purpose:** Public health leaders have advocated for clinical and population-based interventions to address the social determinants of health (SDoH). The American Academy of Family Physicians has worked to support family physicians with addressing the SDoH. However, the extent that family physicians are engaged and the factors that influence this are unknown.

**Methods:** A survey was used to identify actions family physicians had taken to address the SDoH and perceived barriers. Physician and community characteristics were linked. Ordinal logistic regression was used to identify factors associated with engagement in clinical and population-based actions, separately.

**Results:** There were 434 (8.7%) responses. Among respondents, 81.1% were engaged in at least one clinical action, and 43.3% were engaged in at least one population-based action. Time (80.0%) and staffing (64.5%) were the most common barriers. Physician experience was associated with higher levels of clinical engagement, lower median household income was associated with higher levels of population-based engagement, and working for a federally qualified health center (FQHC) was associated with both.

**Conclusions:** The study provides preliminary information suggesting that family physicians are engaged in addressing the SDoH through clinical and population-based actions. Newer family physicians and those working in FQHCs may be good targets for piloting clinical actions to address SDoH and family physician advocates may be more likely to come from an FQHC or in a lower socioeconomic neighborhood. The study also raises questions about the value family physicians serving disadvantaged communities place on clinical interventions to address the SDoH.

## Introduction

The social determinants of health (SDoH) have a substantial impact on population health, contributing more than behavior, clinical care, and the environment.^1,2^ SDoH are also the primary driver of health inequities.^3^ Many organizations have adopted priorities for addressing the SDoH in response to their large impact on population health and comparatively low level of investment.^4,5^ The American Academy of Family Physicians (AAFP) made health equity a strategic priority in 2017.^6^ It has positioned family physicians as important champions for health equity because they more often provide health care in disadvantaged communities than other medical specialties,^7^ and because they focus on primary care.^8^ The AAFP also created its Center for Diversity and Health Equity, which has developed policies, resources, education, support, and engagement opportunities to help family physicians address the upstream and downstream determinants of health inequities.^9,10^ However, it is unclear how family physicians are working to address the SDoH and what factors impact engagement.

The World Health Organization's conceptual framework for action on the SDoH shows that health inequities manifest from socioeconomic group differences in material circumstances, behavior, and psychosocial factors, which are driven by governmental policies and societal culture and values.^11^ Because of the multilevel nature of the SDoH, public health leaders have called for different types of interventions ranging from those that address individuals' social needs to those that aim to change the structural conditions that impact populations. Family physicians could engage in all these strategies. Models exist to integrate SDoH into primary care by incorporating patient-reported and aggregate community data into electronic health records (EHRs) to improve clinical care and facilitate referrals to social service organizations.^12–14^ However, evidence supporting these types of interventions is mixed. For example, evidence-based recommendations exist for community health workers,^15^ but not for social needs screening.^16–18^ In addition, with the advent of strategies like community-based strategic planning and health in all policies, there may be a role for physicians to play in shaping public policy.^19–22^

To inform their work, the AAFP conducted a series of environmental scans,^23^ including this survey. The purpose was to measure the extent that family physicians are engaged in addressing the SDoH. This information may also be useful to organizations that want to engage family physicians in efforts to address the SDoH. This study addressed the following questions:

1. To what extent are family physicians engaged in clinical and population-based actions to address the SDoH?
2. What are family physicians' perceived barriers to addressing the SDoH?
3. What factors are associated with family physicians' level of engagement in clinical and population-based actions to address the SDoH?

## Methods

A multimodal survey was used and was deemed exempt by AAFPs institutional review board.

### Questionnaire and data sources

The questionnaire was developed by a team of family physicians, survey researchers, epidemiologists, and health care and public health managers. The questionnaire was developed based on a literature review about clinical and population-based interventions to address the SDoH (referred to as clinical actions and population-based actions from this point forward).^12,13,16,17,24–29^ Input from 16 family physicians was also used from the AAFPs Subcommittee on Health Equity.

The outcomes of interest were clinical and population-based actions that were separated because of their distinct research threads. The medical literature focuses more on clinical interventions and the public health literature focuses more on population-based interventions. Clinical interventions were addressed using five yes/no statements. Participants were asked, “Which of the following do you participate in or do on a regular basis in your practice?” Statements included the following: screen patients for SDoH, refer patients to community-based resources to address SDoH, capture SDoH data in EHRs, use community health workers to address patient's SDoH, and use community health data to complement patient information. Population-based interventions were addressed using three yes/no statements. Participants were asked, “Which of the following have you engaged in to address social determinants of health?” Statements included the following: written or spoken with elected officials to support public policies aimed at addressing SDoH; provided testimony at a hearing to support public policies aimed at addressing SDoH; and were involved in community health needs assessment, community health improvement planning (CHIP), or other collaborative initiatives aimed at improving community health.

Barriers were addressed using eight yes/no statements. Participants were asked, “Which of the following are barriers to identifying and helping to address your patients' social determinants of health?” Statements included the following: time; staffing; ability to provide solutions; financial incentives; community resources; education or training on SDoH; resources integrated in EHRs; and evidence to support how to address SDoH. Barriers rather than facilitators were focused on because family physicians suggested that there were more clear barriers. Open-ended questions were not included because of the perceived respondent burden. Pilot testing was conducted with two family physicians and the survey was finalized when no significant recommendations for modification were provided.

Independent variables were included to measure physician and community characteristics. Physician characteristics included the following: sex; years since residency; employment status; primary patient care location; as well as the type of disciplines employed at the practice, including clinical support staff, nurse practitioners, physician assistants, social workers, health educators, and community health workers. This information was obtained from the AAFPs member census and linked to the survey.^30^ Community characteristics were included to examine whether socioeconomic aspects of the communities family physicians worked impacted their engagement beyond their own characteristics.^31^ Median household income was included as a general measure of the average socioeconomic position of the community. Lower median incomes represented lower socioeconomic status.^31^ The Gini coefficient was included as a measure of income inequality. Lower scores represented more equality.^32^ The social deprivation index was included as a composite measure made up of single parent families, poverty, percent with less than a high school diploma, unemployment, and car ownership.^33^ Lower scores represented less deprivation. Practice locations were geocoded and relevant geographic identifiers were assigned.^34^ Information about community characteristics were obtained from the 2011–2015 American Community Survey Five-Year Estimates Program and appended to each practice at the zip code tabulation area (ZCTA) level of geography.^35^ ZCTAs were selected as the proxy of the physician's community because previous research indicated that zip codes are the most common catchment area for primary care practices.^36,37^

### Data collection

A randomly selected sample of 5000 practicing physician members of the AAFP were recruited. Invitees were sent a printed copy by mail and a web survey by e-mail in July 2017. The web survey was sent again in September 2017. A $50 incentive was offered to individuals that completed the survey.

### Data analysis

Stata version 15 was used for data analysis.^38^ The proportion was calculated for each of the variables. Two outcome variables were created to measure the level of family physician engagement in clinical and population-based actions, separately. Clinical engagement was based on the sum of the clinical actions (range=0–5). Population-based engagement was based on the sum of population-based actions (range=0–3). Lower values represented less engagement.

Stata's multiple imputation commands were used because 14.3% of participants were missing data. Among the participants missing data, 98% were missing three or fewer variables. Complete case analysis was found to be inappropriate because the missing completely at random assumption was violated.^39^ This was assessed by testing the association of missing or not missing data with each variable. Missing data were associated (*p*<0.05) with years since residency and primary patient care location. The imputation process included all variables included in the analysis and 30 imputed data sets were generated.

Ordinal logistic regression was conducted yielding crude and adjusted odds ratios (AORs) to assess for the effect of physician and community characteristics on the level of engagement in clinical and population-based actions. Crude odds ratios were examined for each variable. Variables with *p*≤0.10 were included in the multivariable model, based on model building strategies from Heeringa, West, and Berglund.^40^ The proportional odds assumption was tested for each crude and multivariable model.

## Results

### Physician and community characteristics

There were 434 (8.7%) valid responses to the survey. All participants worked in a health care setting. Sixty-nine percent were employed by their practice and 31% owned their own practice. Most participants also had seven or more years of experience since residency (84.3%). Sex was approximately evenly distributed with 58.5% being men and 41.5% being women. Most participants provided care in an office or clinic setting (78.8%), with fewer providing care in a hospital (11.3%) or a federally qualified health center (FQHC) (9.0%). A majority of participants employed clinical support staff (85.3%) and nurse practitioners (57.6%). Fewer than half employed physician assistants (40.3%), social workers (25.1%), health educators (20.0%), and community health workers (9.7%). Participants worked in a variety of communities. Nine percent of participants worked in a ZCTA with a median household income of <$35,000, compared with 17.1% for $35,000–$44,999, 28.8% for $45,000–$59,999, 10.6% for $60,000–$69,999, and 21.4% for ≥70,000. Twelve percent of participants worked in a ZCTA with a Gini coefficient <0.40, compared with 35.3% for 0.40–0.44, 24.2% for 0.45–0.49, and 16.1%≥0.50. About 16.1% of participants worked in a ZCTA with a social deprivation index less than −1.00, compared with 32.5% for −0.99 to 0.00, 27.9% for 0.01–0.99, and 10.6% for ≥1 (Table 1).

**Table 1.** Characteristics of the Survey Sample and Sampling Frame

Characteristics	Survey	Sampling frame^a^
	% (95% CI)	%^b^
Sex		
Male	58.5 (53.8–63.1)	56.5
Female	41.5 (36.9–46.2)	43.5
Physician experience		
≤7 Years since residency	84.3 (80.6–87.5)	25.0
>7 Years since residency	15.7 (12.5–19.4)	75.0
Practice ownership		
Owner	30.6 (26.5–35.2)	28.0
Employed	69.4 (64.8–73.5)	68.0
Primary patient care setting		
Office or clinic	78.8 (74.7–82.4)	75.0
Federally Qualified Community Health Center	9.0 (6.6–12.1)	9.0
Hospital	11.3 (8.6–14.6)	7.0
Employ clinical support staff (yes)	85.3 (81.6–88.3)	
Employ nurse practitioners (yes)	57.6 (52.9–62.2)	
Employ physician assistants (yes)	40.3 (35.8–45.0)	
Employ social workers (yes)	25.1 (21.2–29.4)	
Employ health educators (yes)	20.0 (16.3–23.9)	
Employ community health workers (yes)	9.7 (7.2–12.9)	
Median household income of ZCTA		
<$35,000	9.2 (6.8–12.3)	
$35,000–$44,999	17.1 (13.8–20.9)	
$45,000–$59,999	28.8 (24.7–33.3)	
$60,000–$69,999	10.6 (8.0–13.9)	
≥$70,000	21.4 (17.8–25.6)	
Gini coefficient of ZCTA		
<0.40	11.5 (8.8–14.9)	
0.40–0.44	35.3 (30.9–39.9)	
0.45–0.49	24.2 (20.4–28.5)	
≥0.50	16.1 (12.9–19.9)	
Social deprivation index in ZCTA		
<−1.00	16.1 (12.9–19.9)	
−0.99–0.00	32.5 (28.2–37.1)	
0.01–0.99	27.9 (23.8–32.3)	
≥1.00	10.6 (8.0–13.9)	

Sample *N*=434. Sampling frame *N*=68,921. Because of missing data, categories may not sum to 100%.

^a^ The American Academy of Family Physician 2017 Member Census.

^b^ Information on the CIs was not available from the report. Not all values for the sampling frame total to 100% because some categories captured in the sampling frame were not applicable to this survey.

AAFP, American Academy of Family Physicians; CI, confidence interval; ZCTA, zip code tabulation area.

### Engagement in actions to address the SDoH

Participants were engaged in a variety of clinical and population-based actions. At the clinical level: 58.8% screened patients for SDoH, 51.2% referred patients to community-based resources to address SDoH, 41.9% captured SDoH data in their EHRs, 33.4% used community health workers to address patients' SDoH, and 18.9% used community health data to complement patient information. At the population-level 25.8% had written or spoken with elected officials to support public policies aimed at addressing SDoH, 5.5% had provided testimony at a hearing to support public policies aimed at addressing SDoH, and 31.6% were involved in community health needs assessment, CHIP, or other collaborative initiatives aimed at improving community health. Participants were skewed toward lower levels of engagement for clinical and population-based activities to address SDoH (Table 2).

**Table 2.** Clinical and Population-Based Actions to Address the Social Determinants of Health

Actions to address the social determinants of health	% (95% CI)
Clinical actions	
Screen patients for SDoH (yes)	58.8 (54.0–63.3)
Refer patients to community-based resources to address SDoH (yes)	51.2 (43.9–53.3)
Capture SDoH data in EHR (yes)	41.9 (37.4–46.7)
Use community health workers to address patient's SDoH (yes)	33.4 (29.1–38.0)
Use community health data to complement patient information (yes)	18.9 (15.5–22.9)
Level of engagement in clinical actions	
0 Activities	18.9 (15.5–22.9)
1 Activity	19.4 (15.9–23.4)
2 Activities	22.4 (18.7–26.5)
3 Activities	22.8 (19.1–27.0)
4 Activities	9.9 (7.4–13.1)
5 Activities	6.5 (4.5–9.2)
Population-based actions	
Communicated with elected officials to support policies for SDoH (yes)	25.8 (21.9–30.1)
Provided testimony to support policies for SDoH (yes)	5.5 (3.7–8.1)
Involved in collaborative community health initiatives (CHA, CHIP, etc.) (yes)	31.6 (27.3–36.1)
Level of engagement in population-based activities	
0 Activities	56.7 (52.0–61.3)
1 Activity	25.8 (21.9–30.1)
2 Activities	14.1 (11.1–17.7)
3 Activities	3.0 (1.7–5.1)

*n*=434.

CHA, community health assessment; CHIP, community health improvement planning; EHR, electronic health record; SDoH, social determinants of health.

### Barriers

More than half of the study participants stated that time (80.0%), staffing (64.5%), ability to provide a solution (55.5%), and lack of financial incentives (53.0%) were barriers. A smaller but still substantial number of participants stated that lack of either resources in their community (44.2%), education or training (40.8%), or resources integrated in EHRs (36.6%) were barriers. Relatively few participants said that there was a lack of evidence to support addressing the SDoH (13.8%) (Table 3).

**Table 3.** Barriers Faced by Family Physicians to Act on the Social Determinants of Health

Barriers and facilitators	% (95% CI)
The time it takes to discuss the topic with patients (yes)	80.0 (75.9–83.5)
Not properly staffed to address social determinants of health (yes)	64.5 (60.0–68.9)
Inability to provide a solution for my patients (yes)	55.5 (50.8–60.2)
Lack of financial incentives to address SDoH (yes)	53.0 (48.3–57.7)
Lack of resources in my community (yes)	44.2 (39.6–49.0)
Lack of education or training on the topic (yes)	40.8 (36.2–45.5)
Lack of resources integrated with EHRs (yes)	36.6 (32.2–41.3)
Lack of evidence to support social determinants of health (yes)	13.8 (10.9–17.4)

*n*=434.

### Factors predicting engagement

After adjusting for covariates, family physicians with ≤7 years of experience since residency (AOR=2.13, 95% confidence interval [CI]=1.33–3.40) were more engaged in clinical actions than those with more experience. In addition, family physicians who worked in an FQHC (AOR=2.10, 95% CI=1.11–3.96) were more engaged in clinical actions, and family physicians who worked in a hospital setting were less engaged (AOR=0.56, 95% CI=0.32–0.96) compared with those who worked in an office or clinic setting. Community characteristics were not associated with the level of engagement in clinical actions.

After adjusting for covariates, family physicians who worked in an FQHC (AOR=3.90, 95% CI=1.96–7.66) were more engaged in population-based actions than those that worked in an office or clinic setting. In addition, family physicians who worked in ZCTAs with a median household income of <$35,000 (AOR=4.71, 95% CI=1.63–13.65) and $35,000 to $44,999 (AOR=2.93, 95% CI=1.18–7.25) were more engaged in population-based actions compared with those who worked in ZCTAs ≥$70,000 (Table 4).

**Table 4.** Association of Family Physician and Community Characteristics with the Level of Engagement in Clinical and Population-Based Actions to Address the Social Determinants of Health

Independent variables	Clinical actions				Population-based actions			
	Crude analysis		Adjusted analysis		Crude analysis		Adjusted analysis	
	OR	95% CI	AOR	95% CI	OR	95% CI	AOR	95% CI
Gender (female/male)^a^	1.22	0.87–1.71			1.02	0.70–1.48		
Physician experience								
>7 Years of experience	—	—	—	—	—	—	—	—
≤7 Years of experience	2.46^***^	1.56–3.88	2.13^**^	1.33–3.40	1.72^*^	1.07–2.75	1.38	0.81–2.35
Practice ownership								
Owner	—	—	—	—	—	—	—	—
Employed	1.75^**^	1.22–2.53	1.32	0.89–1.94	1.71^*^	1.13–2.58	0.96	0.60–1.53
Employ clinical support staff^b^	1.78^*^	1.10–2.86	1.41	0.84–2.35	1.51	0.88–2.50		
Employ nurse practitioners^b^	1.37	0.98–1.91			1.49^*^	1.02–2.17	1.09	0.70–1.69
Employ physician assistant^b^	1.26	0.89–1.77			1.52^*^	1.05–2.20	1.18	0.77–1.82
Employ social workers^b^	2.23^***^	1.51–3.30	1.44	0.92–2.25	2.61^***^	1.74–3.93	1.56	0.94–2.59
Employ health educators^b^	1.91^**^	1.24–2.93	1.34	0.84–2.13	2.19^***^	1.41–3.40	1.47	0.86–2.48
Employ community health workers^b^	2.70^***^	1.52–4.80	1.51	0.79–2.87	2.66^***^	1.48–4.77	0.94	0.44–1.98
Primary patient care location								
Office or clinic	—	—	—	—	—	—	—	—
FQHC	3.18^***^	1.77–5.73	2.10^*^	1.11–3.96	5.63^***^	3.04–10.40	3.90^***^	1.96–7.66
Hospital	0.68	0.41–1.15	0.56	0.32–0.96	2.02^*^	1.14–3.59	1.46	0.79–2.73
Median household income in ZCTA								
≥$70,000	—	—			—	—	—	—
$60,000–$69,999	0.95	0.49–1.84			1.66	0.79–3.49	1.54	0.66–3.58
$45,000–$59,999	0.76	0.48–1.21			1.65	0.93–2.91	1.56	0.74–3.31
$35,000–$44,999	0.91	0.53–1.56			3.17^***^	1.70–5.90	2.93^*^	1.18–7.25
<$35,000	1.00	0.51–1.97			7.63^***^	3.58–16.25	4.71^*^	1.63–13.65
Gini coefficient in ZCTA								
<0.40	—	—			—	—	—	—
0.40–0.44	1.56	0.90–2.70			1.56	0.83–2.96	1.40	0.69–2.81
0.45–0.49	1.38	0.76–2.48			1.36	0.68–2.70	0.86	0.39–1.86
≥0.50	1.53	0.81–2.90			2.42^*^	1.20–4.87	1.44	0.50–2.68
Social deprivation index in ZCTA								
<−1.00	—	—			—	—	—	—
−0.99–0.00	0.94	0.56–1.57			1.30	0.69–2.48	0.89	0.40–1.96
0.01–0.99	0.86	0.51–1.43			2.27^*^	1.19–4.38	1.04	0.40–2.68
≥1.00	1.53	0.75–3.13			5.41^***^	2.47–11.83	1.44	0.50–4.67

Ordinal logistic regression was used to calculate crude and AORs, 95% CIs, and *p*-values for the association of the independent variables with the level of engagement in clinical actions and population-based actions to address the social determinants of health separately. *N*=434, multiple imputation was used to generate 30 imputed datasets; ^*^p<0.05; ^**^p<0.01; ^***^p<0.001.

^a^ Reference is male.

^b^ Reference is not employing this type of staff member.

AOR, adjusted odds ratio; FQHC, federally qualified health center; OR, odds ratio.

## Discussion

This study provides important preliminary information about the role that family physicians play in addressing SDoH. The findings generate several hypotheses and raise questions for further study. However, limitations may threaten the validity of these findings.

### Family physician engagement for SDoH

Most participants were engaged in at least one type of clinical action. Screening, referral, and use of EHRs were most commonly used. Few participants used community health data. Frameworks, such as “community vital signs,” exist to integrate these aspects of care.^14^ However, the findings suggest that incorporating community health data may be an impediment to operationalizing these frameworks, because of their low uptake. The data also suggest that newer physicians and physicians working in FQHCs may be earlier adopters and targets for piloting these types of frameworks. Opportunities may also exist to expand the use of community health workers because there are recommendations for their effectiveness.^15^

A smaller, but still substantial, number of participants were engaged in population-based actions. This is important because community-based strategic planning for health^22^ and health in all policies^21^ are being recognized as key strategies for addressing the structural drivers of health inequities. Family physicians could be important advocates for health equity. Although the proportion of family physicians partaking in community engagement and advocacy initiatives may appear too high, it is in line with a 2006 study by Gruen et al. that showed that a substantial number of family physicians who participated in their community were politically active and advocated with their professional society.^20^ The data suggest that family physicians who work for an FQHC or work in communities with lower socioeconomic status may be more likely advocates for health equity.

### Issues affecting engagement

The most substantial barriers reported by participants from the options that were presented were related to organizational capacity (time, financial incentives, and staff).^41^ Payment reform could provide the funding mechanisms to support SDoH in primary care, but there is currently insufficient evidence to support many of these practices.^18^ Additional research is needed to verify the effectiveness of these types of initiatives to influence payment systems. Participants also reported that there were insufficient resources in their communities to provide their patients with useful solutions. This problem may have been exacerbated by recent funding reductions for housing, public health, and other social service programs.^42,43^ These types of organizations need stable and sufficient financing to increase their capacity, improve their effectiveness, and facilitate easier collaboration.^44–46^

Family physicians who worked for an FQHC were more engaged in both clinical and population-based actions. Although not surprising, this highlights the important work of FQHCs. It also raises questions about what FQHC practices could be adopted in other clinical settings, and how FQHCs can be leveraged more for nonclinical community health initiatives.

Family physicians with fewer years of experience were more engaged in clinical actions, as compared with their more experienced colleagues. This could suggest that changes to medical education, such as the Population Health Milestones in Graduate Medical Education, have motivated and equipped newer family physicians to address the SDoH.^47^ The Affordable Care Act may have also created the need for these interventions because access to health care was expanded. In addition, technological changes may have helped facilitate these interventions as EHR adoption has become more widespread, and new technology like Aunt Bertha and 211 have helped facilitate referrals to community-based resources.^48–50^

The findings also showed that family physicians who worked in communities with lower socioeconomic status were more engaged in population-based actions than their peers who worked in more advantaged communities. However, community characteristics did not influence clinical engagement. This was surprising with family medicine having a strong focus on the patient and physician relationship.^51^ Editorials by Hollander-Rodriguez and DeVoe, and by Sikora and Johnson call for family medicine to be accountable for population health because the specialty places the health of patients in the context of family and community and because the health of the broader community affects the health of individuals.^52,53^ This raises questions about how family physicians who work with disadvantaged communities view the benefit of clinical actions for SDoH in comparison with more population-based interventions. Family physicians serving disadvantaged communities may lack the resources they need to implement clinical interventions because there are disparities to access care and health insurance.^54^ It could also mean that they see more value in addressing the root structural causes of health inequities.^55^ More research may be needed on this topic to understand how to engage family physicians.

### Limitations

There were several limitations. The response rate was low and less experienced physicians were overrepresented, which may have inflated the observed engagement of family physicians. Engagement was operationalized as the sum of a selection of actions to address SDoH and did not consider factors like dedicated time or resources. This survey was limited to AAFP members and may not be generalizable to the full population of family physicians in the United States. Moreover, 14.3% of respondents were missing data. Multiple imputations were used to address this, but the results may still be prone to bias.^56^ Finally, information was based on self-report alone and causality cannot be determined from cross-sectional surveys. Although the limitations may raise questions about the validity of the findings, the study was intended to provide preliminary data on this topic to allow for a baseline assessment and to identify areas for further inquiry.

## Conclusion: Implications for health equity

This study provides preliminary data about family physicians' engagement in addressing SDoH. The findings suggest that some family physicians are using clinical interventions and population-based strategies to address the SDoH. However, the level of engagement varied, and several barriers were identified. Organizational capacity of medical practices was a major issue and having the financial resources to support the number and type of staff needed to apply clinical interventions for SDoH seems problematic. The organizational capacity of community-based organizations and social services may also be an issue affecting their ability to collaborate with primary care.^57^ Family physicians who worked for FQHCs were more engaged in both clinical and population-based actions. Further study may be needed to determine if and how FQHCs incentivize this work. Newer family physicians were more engaged in clinical activities. There are many potential reasons for this that warrants additional study. Finally, family physicians who worked in disadvantaged communities were more likely to be engaged in population-based actions, but not clinical actions. This raises a variety of questions about the family physicians' perceived value of clinical interventions for SDoH.

## Abbreviations Used

AAFP: American Academy of Family Physicians
AOR: adjusted odds ratio
CHA: community health assessment
CHIP: community health improvement planning
CI: confidence interval
EHR: electronic health record
FQHC: federally qualified health center
OR: odds ratio
SDoH: social determinants of health
ZCTA: zip code tabulation area
